# Effects of providing access to a cycling exergaming platform on cardiorespiratory fitness and cardiometabolic health in adolescents: a randomised, controlled trial

**DOI:** 10.1038/s41598-026-48327-3

**Published:** 2026-05-08

**Authors:** Jonathan Berg, Alf Inge Wang, Erik Madssen, Lisbeth Røe, Trine Moholdt

**Affiliations:** 1 Department of Circulation and Medical Imaging, Norwegian University of Science and Technology, Trondheim, Norway; 2 Department of Cardiology and Cardiothoracic surgery, St. Olav’s University Hospital, Trondheim, Norway; 3 Department of Computer Science, Norwegian University of Science and Technology, Trondheim, Norway; 4 Department of Gynaecology and Obstetrics, St. Olav’s University Hospital, Trondheim, Norway

**Keywords:** Active videogames, Cardiorespiratory fitness, Inactivity, Obesity, Physical activity, Technology, Risk factors, Randomized controlled trials

## Abstract

Insufficient physical activity (PA) among adolescents has deleterious consequences for their health. Exergaming, which combines exercise with gaming, is enjoyable, but the long-term effects of exergaming on adolescent health remain poorly understood. In this randomised, controlled trial, we examined the impact of 24-week access to a cycling exergaming platform versus a non-intervention control group on peak oxygen uptake (V̇O_2peak_), PA levels, and other cardiometabolic outcomes in healthy 12-18-year-olds not engaged in regular endurance training. Twenty-nine participants were randomly assigned to either the exergaming (*n* = 13) or control (*n* = 16) group. There was no statistically significant effect of the 24-week intervention on V̇O_2peak_ (2.4 mL·kg^−1^·min^−1^ [95% CI, -1.6 to 6.5, *p* = 0.23]). Still, compared to the control group, the exergaming group showed improvements in BMI (-1.3 kg/m^2^ [95% CI, -0.4 to -2.2, *p* = 0.007]), body fat mass (-3.6 kg [95% CI, -1.1 to -6.1, *p* = 0.007]), and other adiposity measures. Providing adolescents not engaged in endurance training with access to an exergaming platform did not increase V̇O_2peak_ but did improve body composition. However, the limited sample size, the few exergaming sessions completed, and the underrepresentation of female participants limit the broader applicability of our findings. **Trial registration**: NCT03663699.

## Introduction

Most adolescents worldwide fail to meet the World Health Organisation’s recommended 60 min of daily physical activity (PA)^1^. Since PA behaviours established in adolescence often track into adulthood, and physically active adolescents have lower adiposity, better cardiometabolic health, and higher cardiorespiratory fitness, identifying effective methods to promote PA during adolescence is essential^2,3^.

For adolescents, enjoyment is a crucial motivator for engaging in PA, while lack of time and competing interests are common barriers^4^. While many adolescents participate in organised sports, longitudinal data from Australia show that over one-third drop out between ages 10 and 14, indicating that traditional sports do not engage all young people^5^. In contrast, screen-based activities play a prominent role in most adolescents’ lives, with approximately 75% of 9-18-year-olds in Norway regularly playing computer or console games^6^. Exergaming, the playing of digital games requiring physical effort, leverages the interest in screen-based activities to promote PA^7^. By inducing moderate-to-vigorous exercise intensities and providing higher enjoyment than traditional exercise, exergaming can encourage PA without competing with a highly valued activity^8–10^. However, the intensity of exergaming varies widely, from light to vigorous^11^, meaning the potential health benefits are also variable, with higher-intensity exercise likely offering greater benefits for adolescent health^12^.

A systematic review and meta-analysis reported that exergaming modestly reduces body mass index (BMI) and improves certain body composition parameters in adolescents^13^. However, the two randomised controlled trials (RCTs) in adolescents included in that review did not report exercise intensity^14,15^, and acute studies of the same exergames indicate that they elicit only moderate-intensity exercise (≤ 5 metabolic equivalents)^16,17^. Additionally, in several prior exergaming trials, researchers also imposed predetermined frequency and duration of exergaming sessions^14,18–20^. To establish exergaming as a viable alternative to traditional exercise training, it is essential to determine its effectiveness in real-world settings, where participants engage at their own discretion^10^. Furthermore, although cardiorespiratory fitness is a well-established determinant of adolescent health, previous exergaming studies have relied on indirect or submaximal measures of cardiorespiratory fitness^13,21^. Hence, the effect of exergaming on cardiorespiratory fitness in adolescents, as measured by the gold-standard cardiopulmonary exercise test, remains unknown.

We aimed to investigate the effects of 24 weeks of access to exergaming, using the Playpulse exergaming platform, which can produce vigorous exercise intensities^22^, on cardiorespiratory fitness and other markers of cardiometabolic health in adolescents who do not engage in regular endurance training.

## Materials and methods

### Study design

We conducted an RCT with two parallel groups: an exergaming group and a control group. The trial was undertaken at the Norwegian University of Science and Technology (NTNU) and the St. Olav’s University Hospital in Trondheim, Norway. The Regional Committee for Medical and Health Research Ethics (REK-Midt) in Central Norway approved the study (2018/633). REK-Midt is an independent governmental administrative body organised under the medical faculty at NTNU. The original ethics approval, granted on 15 May 2018, has been formally extended several times and therefore remained valid throughout data collection, data analyses, and manuscript preparation. We pre-registered the trial on https://clinicaltrials.gov/ (NCT03663699). The CONSORT and TiDieR Checklists are provided in Additional Files 1 and 2.

### Recruitment and participants

We recruited participants through public advertisements on the web pages of St. Olav’s Hospital and NTNU, and via social media. To be eligible to participate, individuals had to be between 12 and 18 years old and able to ride a bike for 60 min. Because adolescents who frequently play non-active video games are more likely to play exergames, we required all participants to play non-active video games regularly (> 10 h per week) based on their current habits, to increase their likelihood of engaging with our exergaming platform^23^. We excluded participants with known cardiovascular disease, type I diabetes, and those regularly participating in endurance training. All participants received written information about the study procedures. Participants aged 16–18 signed the consent form with a legal guardian. For participants under 16, two legal guardians signed the consent form.

### Randomisation and blinding

After baseline assessments, we randomly allocated participants (1:1) to an exergaming or a control group, stratified by sex. We used WebCRF3, a computerised random number generator developed and operated by the Clinical Research Unit (Klinforsk) at NTNU and St. Olav’s Hospital, Trondheim, Norway, to randomly assign participants. The project coordinator (JB) performed the randomisation for all participants. The randomisation had various block sizes, and the sequence was concealed until groups were assigned. Neither the participants nor the study personnel were blinded.

### Intervention

We provided participants in the exergaming group access to four Playpulse exergaming stations (detailed below) in our laboratory at St. Olav’s University Hospital for 24 weeks. They could use the exergaming platforms as much as they wanted during the intervention, with no mandatory sessions. Participants decided the duration of their sessions but had to pre-book their visits at least 24 h in advance via an online calendar. Participants made the booking anonymously, but the other participants in the intervention group could see when a platform was booked and join the same exergaming session. A research team member (exercise physiologist or research assistant) supervised all exergaming sessions. We recorded the participants’ heart rate (HR) during all sessions using HR monitors (Polar H10, Polar, Kempele, Finland) and reported the mean and peak values relative to the peak HR obtained during the cardiorespiratory fitness assessment. Data on the duration and number of exergaming sessions were automatically tracked and stored through the Playpulse platform’s cloud service. Participants in the control group received no intervention and were not discouraged from engaging in physical activity.

### The Playpulse exergaming platform

**Fig. 1:**
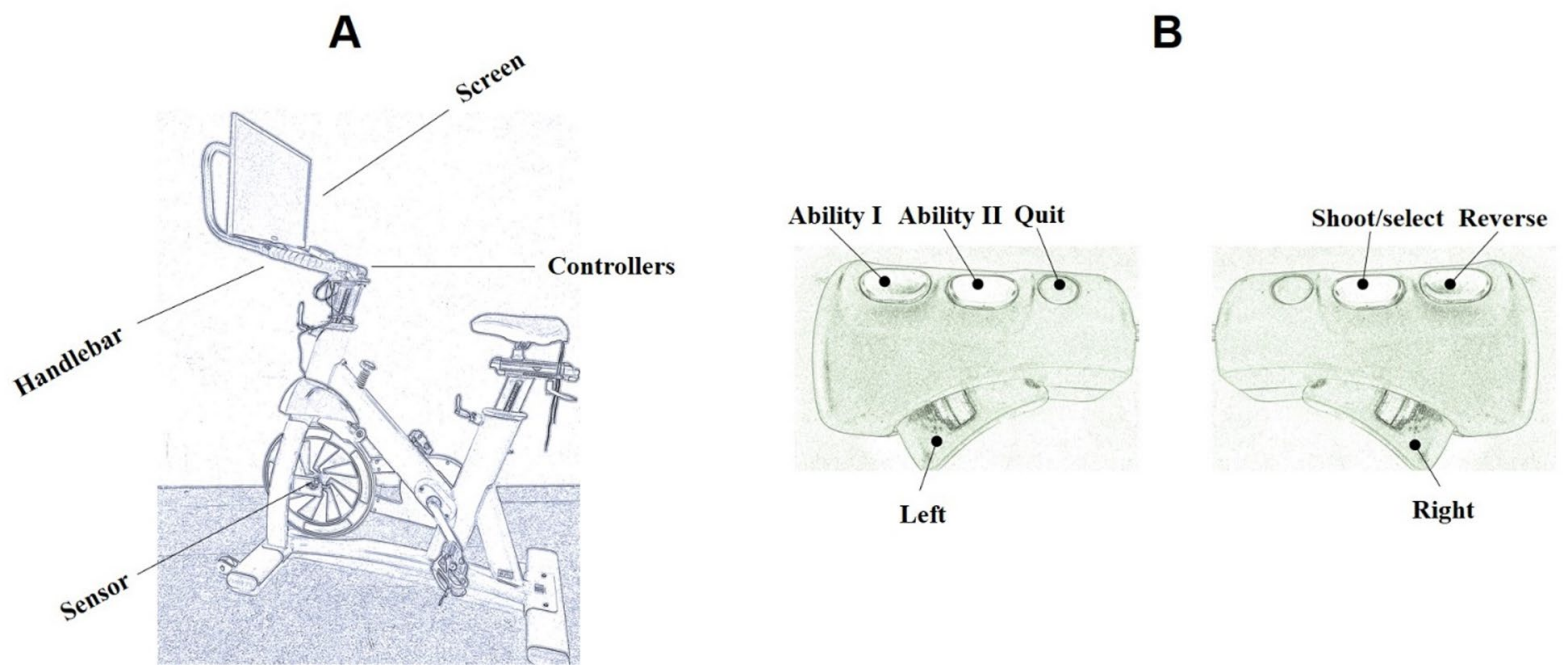
The Playpulse exergaming platform. Illustration of (**A**) the Playpulse exergaming platform with (**B**) buttons on the handlebar. The figure was created with pencilsketch.imageonline.co by Jonathan Berg. Originally published in Frontiers in Sports and Active Living, CC-BY License).

The Playpulse platform comprises a stationary computer and monitor linked to a stationary bicycle ergometer equipped with buttons on the handlebars and a sensor on the front wheel (Fig. 1). With four Playpulse exergaming stations available in our laboratory, we could host up to four participants simultaneously, enabling collaborative exergaming in multiplayer-compatible games. The sensor detects forward bicycle ergometer propulsion, generating movement in the games, whereas the player uses the buttons to control steering and actions. Participants could adjust the resistance on the ergometer, but neither was it monitored nor did it affect gameplay, and no visual feedback on exercise effort was provided. On the Playpulse platform, participants had access to three exergames (Space Race, Bumper Cars, and Pedal Tanks) and could select which to play. In Space Race, which is available in single- and multiplayer modes, the goal is to pedal as fast as possible and manoeuvre various courses to finish first. Bumper Cars can only be played in single-player mode and features different game modes with the overall goal of keeping the car on the track whilst bumping the computer-generated opponents off it. In the multiplayer game Pedal Tanks, the players aim to capture the opponent’s flag and return it to the starting point. Pedal Tanks is described in detail elsewhere^22,24,25^. In all three games, a higher cycling cadence results in greater speed and additional benefits.

### Outcomes

We assessed outcomes on two separate days at baseline, one day after 12 weeks, and two separate days after 24 weeks. On one test day, at baseline and after 24 weeks, blood samples were collected between 9 and 11 a.m. at both timepoints. Participants were instructed to avoid vigorous exercise for two days before the blood sampling day and to fast overnight. On the second test day at baseline and after 24 weeks, and on the single test day after 12 weeks, we assessed anthropometrics, body composition, blood pressure, and cardiorespiratory fitness in that order. Before that testing day, we instructed participants to avoid exercise for ≥ 24 h, caffeine for ≥ 8 h, and fast for ≥ 3 h.

### Cardiorespiratory fitness

The primary outcome was a change in peak oxygen uptake (V̇O_2peak_) relative to body mass (mL·kg^−1^·min^−1^) from baseline to after the intervention (24 weeks). We measured V̇O_2peak_ during a maximal incremental treadmill exercise test (Woodway, Waukesha, WI) using indirect calorimetry (MetaLyzer II, Cortex, Leipzig, Germany). We measured V̇O2peak on a treadmill to reduce the risk of cycling-specific adaptations in exergaming participants and to offer a more familiar exercise mode. The MetaLyzer system was calibrated according to manufacturer recommendations, including ambient-air and volume calibration between tests and daily two-point gas calibration. Following a 10-minute warm-up at a self-selected, moderate intensity, the workload was increased each minute by either 0.5–1 km·h^− 1^ in speed or 1–2% in incline until voluntary exhaustion. Participants chose whether to increase the speed or incline each minute. We calculated V̇O_2peak_ in L·min^−1^ as the average of the three greatest consecutive 10-s values. Then, we scaled V̇O_2peak_ relative to body mass (mL·kg^− 1^·min^− 1^) and fat-free mass (mL·kg_FFM_^−1^·min^− 1^). We recorded HR throughout the test using HR monitors (Polar H10, Polar, Kempele, Finland), with the peak HR observed during the test used as an estimate of maximum HR^26^.

### Body composition and anthropometrics

We measured height to the nearest 0.5 centimetres using a wall-mounted Seca 222 stadiometer (Hamburg, Germany). Body mass, fat mass, fat-free mass, and visceral fat area were estimated using multi-frequency bioelectrical impedance analysis (InBody 720, Biospace, Seoul, South Korea), with participants wearing minimal clothing. We asked participants to void their bladders immediately before the body composition assessment. We did not control for the time of day during follow-up assessments. BMI was calculated as body mass divided by the square of body height and fat mass index as fat mass divided by the square of body height (kg/m^2^).

### Cardiometabolic health

Following 15 min of seated rest, we measured blood pressure three times at 2-minute intervals using an automatic blood pressure device (Philips IntelliVue MP50, Philips Medizin Systeme, Boeblingen, Germany) and report the mean of the final two measurements.

Trained personnel collected venous blood samples from participants after an overnight fast at baseline and after 24 weeks. Samples for plasma-based analyses (glucose, total cholesterol, high-density lipoprotein [HDL] cholesterol, low-density lipoprotein [LDL] cholesterol, and high-sensitivity C-reactive protein [CRP]) were drawn into lithium-heparin tubes. Samples for glycosylated haemoglobin (HbA1c) were collected in EDTA tubes. Plasma was separated according to standardised and accredited procedures at the St. Olav’s Hospital laboratories. Samples were immediately analysed using accredited enzymatic and immunoturbidimetric assays on a Siemens Atellica CH930 analyser, following standardised local protocols. Plasma glucose was measured with an enzymatic photometric assay based on glucose oxidation and NADH formation. Total cholesterol, HDL cholesterol, LDL cholesterol, and triglycerides were determined using enzymatic colourimetric assays, while high-sensitivity CRP was assessed via an immunoturbidimetric assay. HbA1c was measured in EDTA whole blood using an enzymatic method that quantifies the ratio of glycated to total haemoglobin. Additionally, we aliquoted and stored extra blood samples at -80 °C through the Regional Research Biobank at St. Olav’s University Hospital (Biobank1) for future analyses.

### Physical activity monitoring

We fitted participants with activity monitors (SenseWear, BodyMedia, Pittsburgh, Pennsylvania, United States) on their non-dominant upper arm. We asked them to wear the monitors for 24 h, except during showers/baths/saunas, for seven consecutive days before randomisation and after the 12- and 24-week assessments. In the analysis, we included data from participants with at least two days of valid data, defined as any days with ≥ 12 h of data. We report PA as daily means categorised into moderate-, vigorous-, and moderate-to-vigorous intensity PA minutes, with age- and sex-specific intensity thresholds based on median age during the intervention period^27^.

### Protocol changes after study commencement

We originally intended to assess glycaemic control using a 2-h oral glucose tolerance test. However, with a slower-than-anticipated recruitment rate and several interested individuals reluctant to undertake repeated blood sampling, we decided to measure HbA1c to evaluate glycaemic control. Additionally, the prospective trial registration listed insulin as a biomarker but did not include high-sensitivity C-reactive protein (hs-CRP). In the study, insulin was not analysed due to budget constraints, while hs-CRP was collected as part of our routinely planned cardiometabolic biomarker panel. Furthermore, we initially intended to exclude participants who undertook > 60 min of daily moderate-to-vigorous intensity PA, as assessed using activity monitors before inclusion. However, more individuals than expected were excluded for being too active despite reporting inactivity. Therefore, for feasibility, we excluded participants who reported engaging in leisure-time endurance training. All modifications were registered on clinicaltrials.gov and approved by REK-Midt.

### Sample size calculation

Initially, we calculated the sample size to detect a meaningful difference of 3.0 mL·kg^− 1^·min^− 1^ in V̇O_2peak_ at 24 weeks. Assuming a statistical power of 90%, a significance level of 5% for a two-sided independent-samples t-test, and a 15% dropout rate, we determined that we required 39 participants per group. Subsequently, we adjusted the sample size calculation for analysis of covariance (ANCOVA) using the method described by Borm et al.^28^. Based on preliminary data demonstrating a correlation of 0.37 between baseline and 24-week V̇O_2peak_ values, we planned to include 46 participants (23 in each group).

### Statistical analyses

We conducted an intention-to-treat analysis, including all participants with data from at least one time point, using linear mixed models for all outcome variables. By including all available data, linear mixed models provide unbiased estimates under the missing-at-random assumption, whereas complete-case analyses require the more restrictive missing-completely-at-random assumption^29^. In the models, we treated each outcome variable, one at a time, as the dependent variable, with time and the interaction between time and group as fixed effects, and participant (subject ID) as a random effect. The baseline means were constrained to be equal by omitting the group main effect in these models since we expect no systematic baseline differences between groups in RCTs.^30^ The intervention effect was estimated as the group x time interaction and is presented as the between-group difference in change, with corresponding 95% confidence intervals (CIs) and p-values. We assessed the normality of residuals through visual inspection of QQ plots. Variables that did not show normality (V̇O_2peak_ relative to body mass, triglycerides, and high-sensitivity C-reactive protein) were log-transformed. The untransformed data are reported, as log-transformation did not affect the overall findings. For our primary outcome measure (V̇O_2peak_), we considered a *p*-value < 0.05 to indicate statistically significant differences. We used a significance level of < 0.01 for the secondary outcome measures to protect against false-positive results due to multiple comparisons. Furthermore, we performed simple regression analyses using change scores in significant outcome variables from the linear mixed models as dependent variables, and exergaming frequency and intensity as independent variables. All statistical analyses were conducted using IBM SPSS 29.0, and all figures were generated using GraphPadPrism 10.3.1.

### Impact of COVID-19 pandemic

Due to the COVID-19 pandemic, we had to cease all activities in the exercise laboratory, including enrolment, assessments, and exergaming sessions, on 12 March 2020. We informed participants that all activities must end. When the exercise laboratory reopened in August 2020, we invited participants who had had to pause their interventions to repeat the most recent assessments and then resume their previously assigned interventions. Only one of six participants wished to undertake new assessments and continue the intervention. Since the pandemic could have affected lifestyle behaviours, we conducted statistical analyses with and without this participant. Notably, this participant also showed the greatest improvements in body composition. However, since the analyses without the participant did not change the main findings, we report the analyses including the participant.

## Results

### Participants

**Fig. 2:**
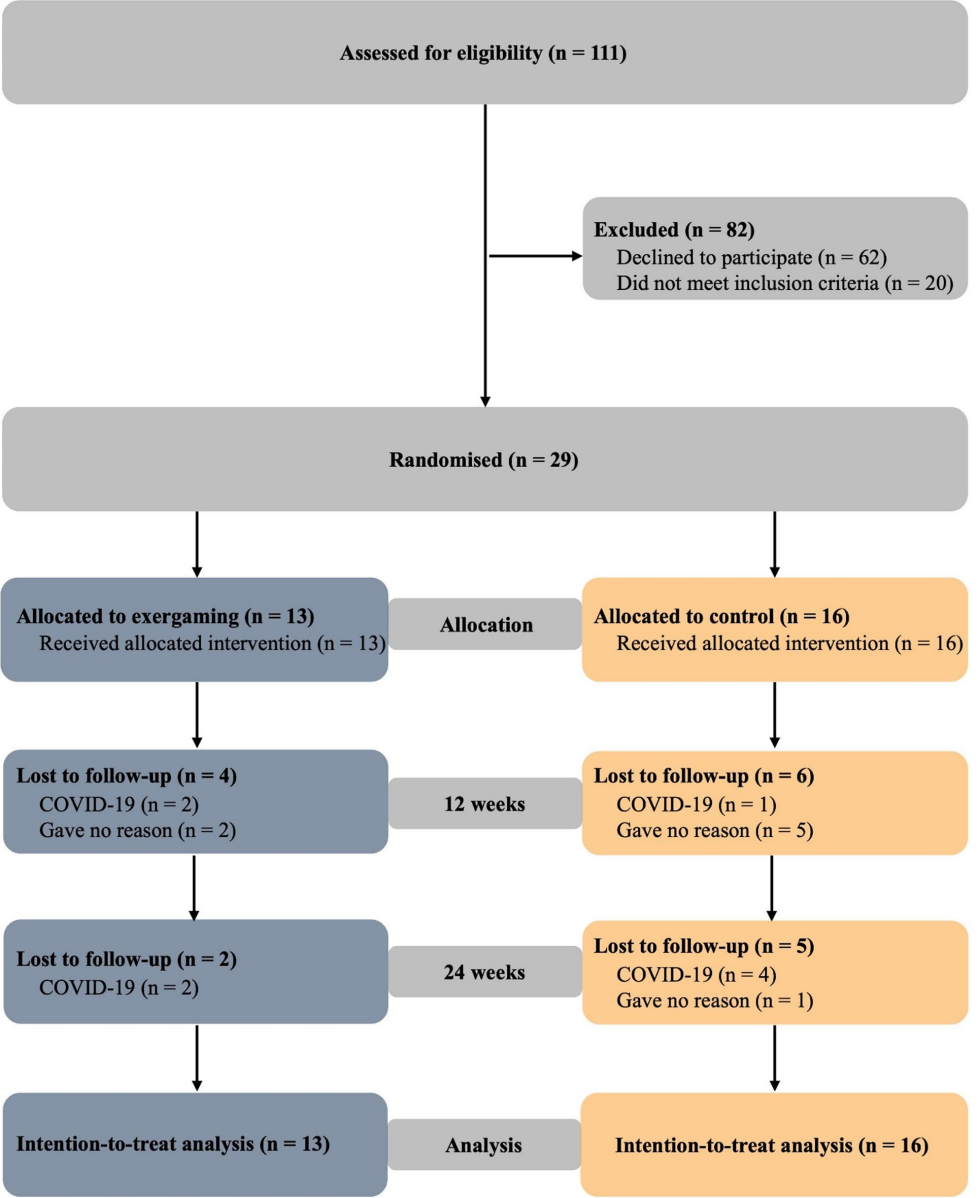
Flowchart of trial. COVID-19: Participants lost to follow-up due to laboratory closures during the COVID-19 lockdown between March and August 2020 or were not interested in continuing participation after the laboratories reopened in August 2020.

We screened 111 interested individuals for eligibility, of whom 29 were randomised (16 to the control group and 13 to the exergaming group) between February 2019 and April 2021 (Fig. 2). We recruited the first participant on 11 February 2019 and completed participant inclusion on 7 April 2021. Although our sample size calculation indicated that a larger sample was needed to detect a clinically relevant difference in V̇O2peak between groups, recruitment difficulties during the study period, where 56% of those initially expressing interest declined to participate, led us to cease recruitment before reaching the target sample size. Seven participants dropped out of the study, six due to the COVID-19 pandemic. Table 1 depicts the baseline characteristics of all randomised participants. We recorded no adverse events during the trial.

**Table 1:** Characteristics of the participants at baseline.

	Control (*n* = 16)	Exergaming (*n* = 13)
Age	14.3 (2.0)	13.0 (1.3)
Height (cm), mean (SD)	169.0 (13.5)	166.5 (12.5)
Body mass index (kg/m^2^), mean (SD)	21.6 (4.3)	22.5 (3.8)
Normal weight, N (%)^a^	11 (68.8)	6 (46.2)
Overweight, N (%)^a^	2 (12.5)	6 (46.2)
Obese, N (%)^a^	3 (18.8)	1 (7.7)
Bodyfat (kg), mean (SD)	13.2 (9.2)	15.9 (7.8)
Bodyfat (%), mean (SD)	19.8 (9.9)	24.2 (8.0)
Visceral fat area (cm^2^), mean (SD)	56.8 (44.6)	71.0 (39.2)
Fat mass index (kg/m^2^), mean (SD)	4.6 (3.2)	5.6 (2.5)
Fat-free mass (kg), mean (SD)	49.1 (11.5)	47.8 (13.1)
Systolic blood pressure (mmHg), mean (SD)	112 (10)	111 (4)
Diastolic blood pressure (mmHg), mean (SD)	69 (7)	70 (5)
High-sensitivity CRP, mean (SD)	0.36 (0.20)	1.54 (2.01)
Missing data, N (%)	1 (6.3)	3 (23.1)
HbA1c (mmol/mol), mean (SD)	32.7 (2.3)	34.3 (3.2)
Missing data, N (%)	1 (6.3)	3 (23.1)
HDL cholesterol (mmol/L), mean (SD)	1.31 (0.29)	1.19 (0.21)
Missing data, N (%)	1 (6.3)	3 (23.1)
LDL cholesterol, mean (SD)	2.3 (0.8)	2.6 (0.7)
Missing data, N (%)	1 (6.3)	3 (23.1)
Total cholesterol, mean (SD)	3.8 (0.7)	3.9 (0.7)
Missing data, N (%)	1 (6.3)	3 (23.1)
Triglyceride level (mmol/L), mean (SD)	0.70 (0.24)	
Missing data, N (%)	1 (6.3)	
Fasting plasma glucose (mmol/L), mean (SD)	4.9 (0.3)	5.0 (0.5)
Missing data, N (%)	1 (6.3)	3 (23.1)
V̇O_2peak_ (L·min^−1^), mean (SD)	2.9 (0.8)	2.8 (0.7)
V̇O_2peak_ (mL·kg^−1^·min^−1^), mean (SD)	47.8 (10.0)	45.2 (6.9)
Healthy V̇O_2peak_, N (%)^b^	11 (68.8)	10 (76.9)
V̇O_2peak_ (mL·kg^FFM−1^·min^−1^), mean (SD)	59.7 (7.6)	59.6 (7.2)
Moderate-to-vigorous intensity physical activity (min·d^− 1^)	67.3 (38.5)	103.0 (58.3)
Physically active, N (%)^c^	5 (31.3)	7 (53.8)
Missing data, N (%)	6 (37.5)	4 (30.8)

C-reactive protein (CRP), glycated haemoglobin (HbA1c), high-density lipoprotein (HDL), low-density lipoprotein (LDL), peak oxygen uptake (V̇O_2peak_), fat-free mass (FFM).

^a^Overweight and obesity are defined as a body mass index passing through 25–30 kg·m^2^ at age 18, respectively^42^.

^b^Healthy peak oxygen uptake defined according to Welk et al.^43^.

^c^Adherence to physical activity guidelines (> 60 min of moderate-to-vigorous intensity physical activity per day).

### Cardiorespiratory fitness

**Fig. 3:**
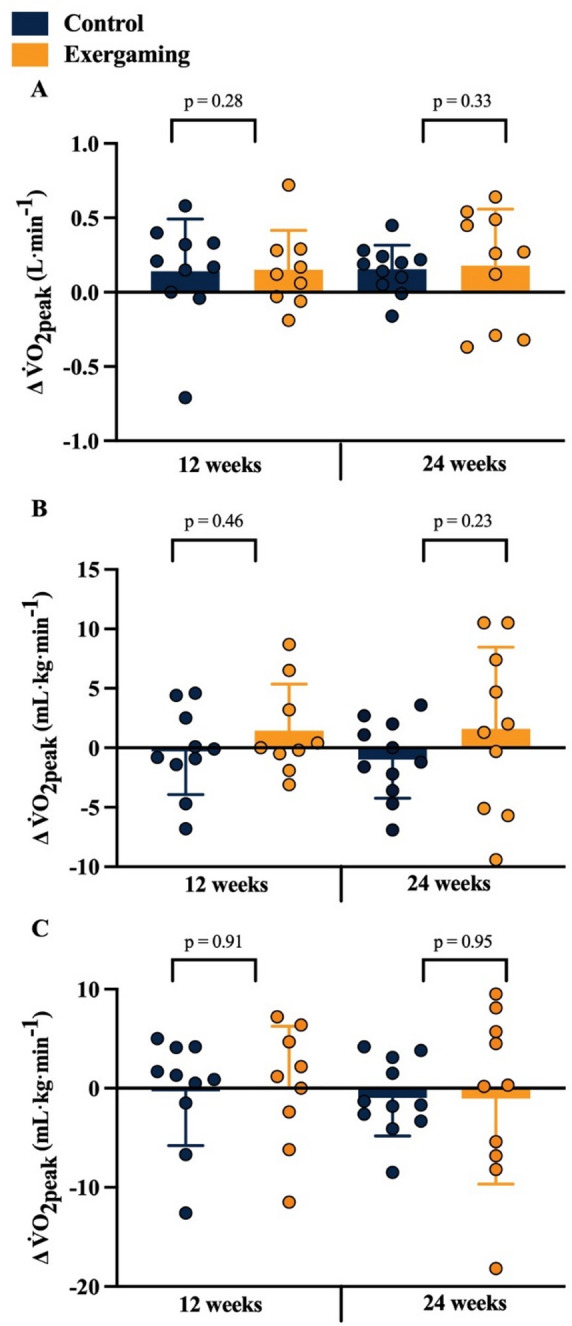
Mean group changes in peak oxygen uptake (V̇O_2peak_) from baseline to 12 and 24 weeks. (**A**) Absolute V̇O_2peak_, (**B**) V̇O_2peak_ relative to body mass, and (**C**) V̇O_2peak_ relative to fat-free mass. All means are descriptive data, with error bars indicating the standard deviation. P-values are for the estimated effects from the linear mixed model. Circles indicate individual observations.

There was no statistically significant effect of 24 weeks of access to an exergaming platform on V̇O_2peak_ relative to body mass (primary outcome measure) (Fig. 3), with an estimated mean difference between groups of 2.4 mL·kg^−1^·min^−1^ (95% CI, -1.6 to 6.5, *p* = 0.23) (Table 2). Nor were there any significant between-group differences after 12 weeks or in other cardiorespiratory fitness outcomes after 12 or 24 weeks of access to the exergaming platform (Table 2; Fig. 3). Peak respiratory exchange ratios were 1.11 (SD, 0.07) and 1.08 (SD, 0.08) at baseline, 1.12 (SD, 0.03) and 1.08 (SD, 0.06) at 12 weeks, and 1.10 (SD, 0.06) and 1.12 (SD, 0.04) for participants in the control and exergaming groups, respectively.

**Table 2:** Values at baseline, 12, and 24 weeks and analyses for primary outcome cardiorespiratory fitness.

			Control		Exergaming	Difference (group x time)		
Outcome	Weeks	N	Mean (SD)	N	Mean (SD)	Estimated effect	95% CI	P value
V̇O_2peak_ (L·min^−1^)	0	16	2.9 (0.8)	13	2.8 (0.7)			
	12	10	3.3 (0.9)	9	3.2 (0.5)	0.0	− 0.5 to 0.3	0.28
	24	11	3.0 (0.8)	10	3.3 (0.6)	0.1	− 0.4 to 0.3	0.33
V̇O_2peak_ (mL·kg^−1^·min^−1^)	0	16	47.8 (10.0)	13	45.2 (6.9)			
	12	10	49.7 (8.1)	9	47.2 (7.9)	1.6	− 2.6 to 5.7	0.46
	24	11	46.5 (9.0)	10	46.7 (6.1)	2.4	− 1.6 to 6.5	0.23
V̇O_2peak_ (mL·kg^FFM−1^·min^−1^)	0	16	59.7 (7.6)	13	59.6 (7.2)			
	12	10	60.8 (6.2)	9	61.2 (7.6)	1.2	− 3.7 to 6.2	0.91
	24	11	57.6 (6.9)	10	59.1 (8.1)	0.9	− 3.9 to 5.6	0.95

Mean (SD) is descriptive data, and the estimated effect is from the linear mixed model. Peak oxygen uptake (V̇O_2peak_).

### Secondary cardiometabolic outcomes

**Fig. 4:**
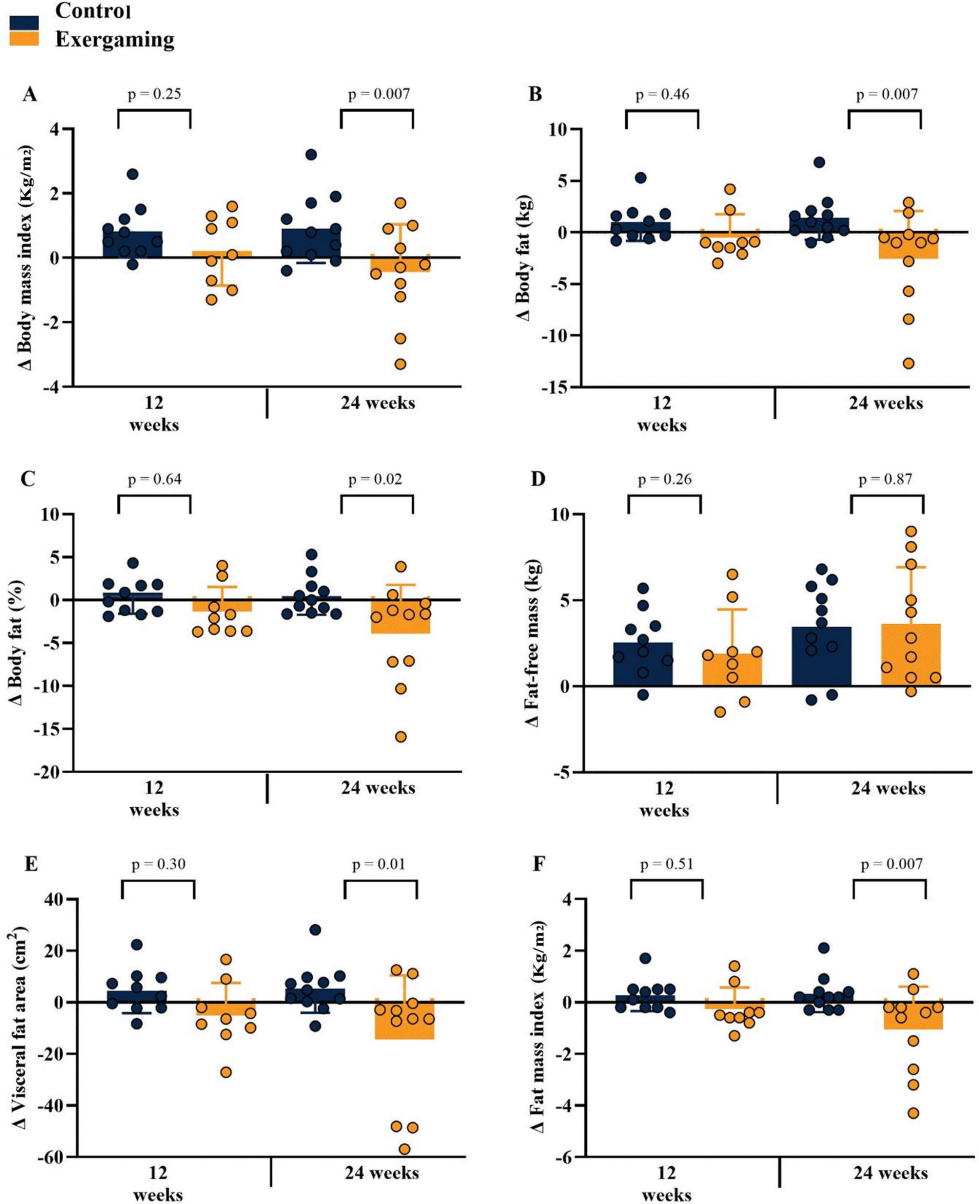
Mean group changes in body mass index and adiposity from baseline to 12 and 24 weeks. (**A**) Body mass index, (**B**) body fat mass, (**C**) body fat percentage, (**D**) visceral fat area, and (**E**) fat mass index. All means are descriptive data, with error bars indicating the standard deviation. P-values are for the estimated effects from the linear mixed model. Circles indicate individual observations.

After 24 weeks, the participants in the exergaming group had reduced BMI, body fat, and fat mass index compared with participants in the control group (Table 3; Fig. 4). There were no significant between-group differences in body composition measures after 12 weeks or for fat-free mass and body fat percentage after 24 weeks (Table 3; Fig. 4). The exergaming group reduced their daily vigorous PA by 7.5 min (95% CI, -12.8 to -2.1, *p* = 0.007) at 12 weeks compared with the control group, with no significant between-group differences for the other PA outcomes at any time point (Table 3). There were no statistically significant differences between the two groups in cardiometabolic health outcome measures (Table 3).

**Table 3:** Values at baseline, 12, and 24 weeks and analyses for secondary outcomes.

			Control		Exergaming	Difference (group x time)		
Outcome	Weeks	N	Mean (SD)	N	Mean (SD)	Estimated effect	95% CI	P value
BMI (kg/m^2^)	0	16	21.6 (4.3)	13	22.5 (3.8)			
	12	10	22.2 (4.4)	9	23.4 (2.9)	− 0.6	− 1.2 to 0.4	0.25
	24	11	22.4 (4.7)	11	23.0 (3.1)	− 1.3	− 0.4 to − 2.2	0.007
Body fat (kg)	0	16	13.2 (9.2)	13	15.9 (7.8)			
	12	10	13.4 (8.8)	9	16.7 (8.3)	− 1.0	− 3.6 to 1.7	0.46
	24	11	13.6 (9.3)	11	14.9 (5.6)	− 3.6	− 1.1 to − 6.1	0.007
Body fat (%)	0	16	19.8 (9.9)	13	24.2 (8.0)			
	12	10	18.6 (8.6)	9	23.5 (9.3)	− 0.7	− 4.0 to 2.5	0.64
	24	11	19.7 (10.0)	11	21.1 (5.9)	− 3.8	− 0.8 to − 6.8	0.02
Fat-free mass (kg)	0	16	49.1 (11.5)	13	47.8 (13.1)			
	12	10	54.5 (12.4)	9	52.0 (9.0)	− 1.4	− 3.7 to 1.0	0.26
	24	11	52.3 (11.6)	11	54.6 (11.7)	0.2	− 2.0 to 2.4	0.87
Visceral fat area (cm^2)^	0	16	56.8 (44.6)	13	71.0 (39.2)			
	12	10	56.4 (41.0)	9	72.7 (40.7)	− 7.7	− 19.2 to 3.8	0.18
	24	11	58.4 (46.4)	11	64.7 (27.7)	− 14.1	− 3.0 to − 25.1	0.01
Fat mass index (kg/m^2^)	0	16	4.6 (3.2)	13	5.6 (2.5)			
	12	10	4.4 (2.7)	9	5.7 (2.7)	− 0.3	− 1.2 to 0.6	0.51
	24	11	4.7 (3.3)	11	5.0 (1.8)	− 1.2	− 0.4 to − 2.1	0.007
Moderate-intensity PA (min·d^− 1^)	0	10	63.1 (37.5)	9	99.2 (56.2)			
	12	7	88.9 (53.6)	6	95.0 (37.0)	6.1	− 44.1 to 56.4	0.81
	24	8	54.3 (36.1)	10	74.0 (38.2)	19.8	− 23.1 to 62.6	0.36
Vigorous-intensity PA (min·d^− 1^)	0	10	4.2 (4.9)	9	3.8 (3.4)			
	12	7	13.7 (7.3)	6	5.7 (2.5)	− 7.5	− 2.1 to − 12.8	0.007
	24	8	3.4 (5.2)	10	4.3 (5.6)	0.8	− 3.9 to 5.4	0.75
Moderate-to-vigorous-intensity PA (min·d^− 1^)	0	10	67.3 (38.5)	9	103.0 (58.3)			
	12	7	102.6 (59.8)	6	100.7 (37.5)	− 1.9	− 55.2 to 51.4	0.94
	24	8	57.6 (40.1)	10	78.3 (41.3)	20.7	− 24.7 to 66.1	0.36
Systolic blood pressure (mmHg)	0	16	112 (10)	13	111 (4)			
	12	10	118 (12)	9	108 (6)	− 7	− 1 to − 14	0.02
	24	11	115 (8)	11	111 (8)	− 3	− 9 to 3	0.28
Diastolic blood pressure (mmHg)	0	16	69 (7)	13	70 (5)			
	12	10	73 (6)	9	67 (6)	− 6	− 2 to − 11	0.01
	24	11	71 (5)	11	68 (5)	− 3	− 7 to 2	0.22
Glucose (mmol·L^− 1^)	0	15	4.9 (0.3)	10	5.0 (0.5)			
	24	6	5.1 (0.4)	5	5.1 (0.1)	− 0.0	− 0.4 to 0.4	0.97
HbA1c (mmol·mol^− 1^)	0	15	33 (2)	10	34 (3)			
	24	6	32 (3)	5	36 (1)	− 0	− 2 to 1	0.65
Total Cholesterol (mmol·L^− 1^)	0	15	3.8 (0.7)	10	3.9 (0.7)			
	24	6	3.5 (0.5)	5	3.8 (0.5)	− 0.1	− 0.6 to 0.3	0.54
HDL Cholesterol (mmol·L^− 1^)	0	15	1.31 (0.29)	10	1.19 (0.21)			
	24	6	1.22 (0.27)	5	1.05 (0.14)	− 0.05	− 0.19 to 0.08	0.40
LDL Cholesterol (mmol·L^− 1^)	0	15	2.3 (0.8)	10	2.6 (0.7)			
	24	6	2.3 (0.6)	5	2.6 (0.6)	− 0.2	− 0.6 to 0.3	0.39
Triglycerides (mmol·L^− 1^)	0	15	0.70 (0.24)	10	1.07 (0.29)			
	24	6	0.75 (0.29)	5	1.14 (0.43)	0.31	− 0.09 to 0.71	0.12
HsCRP (mg·L^− 1^)	0	15	0.36 (0.20)	10	1.54 (2.01)			
	24	6	0.55 (0.32)	5	0.67 (0.82)	0.11	− 1.37 to 1.61	0.88

Mean (SD) is descriptive data, and the estimated effect is from the linear mixed model. Body mass index (BMI, physical activity (PA), glycated haemoglobin (HbA1c), high-density lipoprotein (HDL), low-density lipoprotein (LDL), high-sensitivity c-reactive protein (HsCRP).

### Exergaming attendance, session characteristics and potential predictors of beneficial responses

**Fig. 5:**
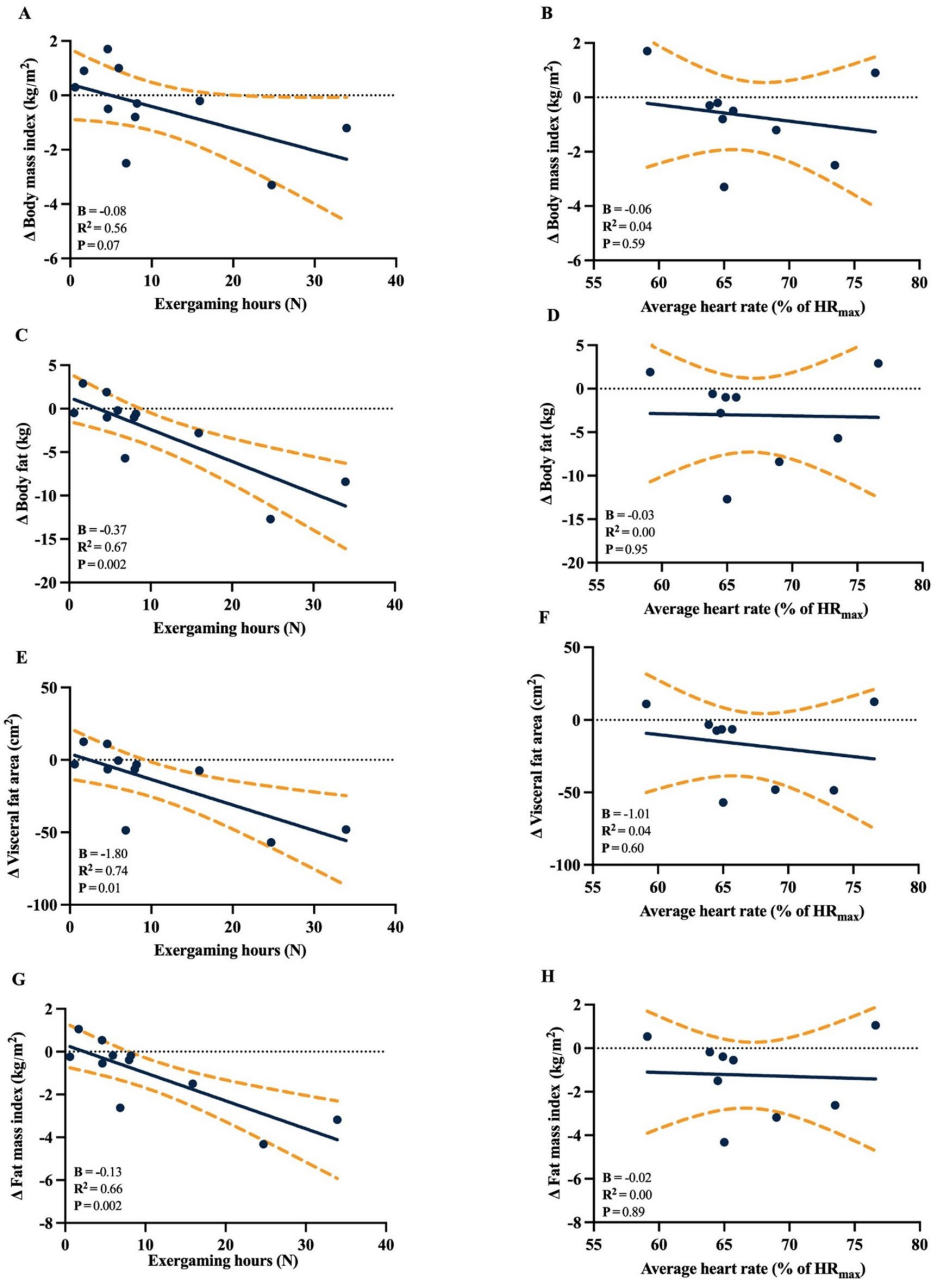
The number of exergaming hours is associated with significant between-group differences in body fat and fat mass index. (**A**) Change in body mass index (BMI), (**C**) body fat mass, (**E**) visceral fat area, and (**G**) fat mass index from baseline to 24 weeks and number of exergaming hours. (**B**) Change in body mass index (BMI), (**D**) body fat mass, (**F**) visceral fat area, and (**H**) fat mass index from baseline to 24 weeks and average exergaming intensity. The dotted yellow lines represent 95% CI.

Over the 24-week intervention period, participants attended 11 exergaming sessions (range, 1 to 39) with an average duration of 45.8 (SD, 12.4) minutes per session. Three participants attended < 6 sessions, 7 attended 6–12 sessions, and 3 attended ≥ 18 sessions. The average and peak exercise intensity for the 78 (53.1%) exergaming sessions with HR data were 67.9% (95% CI, 63.7 to 72.0) and 84.8% (95% CI, 79.4 to 90.1) of maximum HR, respectively. There was a significant relationship between the number of exergaming hours over 24 weeks and reductions in BMI, body fat, visceral fat area, and fat mass index (Fig. 5). However, exercise intensity during exergaming was not associated with changes in body composition outcomes (Fig. 5).

## Discussion

In this 24-week RCT, providing adolescents not involved in regular endurance training with access to a cycling exergaming platform did not improve cardiorespiratory fitness. However, participants in the exergaming group showed more favourable changes in BMI, body fat percentage, visceral fat area, and fat mass index than the control group. The number of exergaming hours was associated with reductions in adiposity, meaning that the more active ‘exergamers’ experienced greater improvements in body composition.

Contrary to our hypothesis, the intervention did not improve cardiorespiratory fitness. Our results agree with a previous study by Maddison et al.^15^, who showed no effect of access to an active video game for 24 weeks on estimated V̇O_2peak_ in 10-14-year-old children with overweight or obesity. Also, in inactive adults, we found no effects on cardiorespiratory fitness after 6 months of access to the same exergaming platform as in the current study due to the low frequency of exergaming^31^. For adolescents, 30–60 min of endurance training three to four times weekly is optimal to improve V̇O_2peak_^32^. Furthermore, intensity must exceed 80% of HR_max_ to significantly impact cardiorespiratory fitness^32^. In contrast to our previous trials using the same exergaming platform in adults, where the average intensity during exergaming ranged from 74 to 80% of HR_max_^22,31,33^_,_ the average intensity in this study was only 68% of HR_max_. Thus, the low exergaming frequency and the exercise intensity during exergaming are likely explanations for the absence of cardiorespiratory fitness benefits. Furthermore, although only adolescents not engaged in endurance training were included, the majority exhibited healthy cardiorespiratory fitness at baseline. This relatively high fitness level, combined with low frequency of exergaming, may have reduced the participants’ potential for improvement in cardiorespiratory fitness. Although not statistically significant, the estimated mean difference between groups of 2.4 ml·kg^−1^·min^−1^ corresponds to ~ 5% relative improvement, which is within the expected range after ≤ 6 months of endurance training in adolescents^32^.

Despite low exergaming attendance, participants in the exergaming group showed greater improvements in BMI and most adiposity measures than the control group, with greater benefits for those who engaged in more exergaming during the intervention period. The estimated mean differences in BMI (-1.3 kg/m²) and body fat (-2.9 kg) after exergaming were greater than those reported by Maddison et al.^15^. Although our observed BMI reduction is modest, the European Society of Endocrinology and the Pediatric Endocrine Society consider a 1.5 kg/m² reduction in BMI achieved through lifestyle interventions in adolescents as clinically meaningful due to its potential long-term health impact^34^. Our findings of reduced adiposity among adolescents align with conclusions from a systematic review and meta-analysis showing improvements in body composition after exergaming in children and adolescents^35^. Notably, improvements in BMI and body composition between groups occurred despite very low exergaming attendance and without between-group changes in objectively measured PA. Thus, the mechanisms underlying these changes remain unclear and future studies designed to capture relevant mechanistic data are needed to address this question. Given the overall low exergaming attendance and no changes in PA, the reasons for the effects on BMI and body composition remain uncertain and raise questions about whether exergaming might induce other lifestyle changes. Maddison et al.^15^ speculated that such changes after exergaming could result from decreased intake of snacks and sweets and from less time spent on sedentary gaming. We, however, did not record our participants’ dietary intake.

Despite the improved body composition after the intervention period, we observed no beneficial effects of exergaming on PA levels. These findings align with a systematic review and meta-analysis of RCTs investigating the impact of active video games on obesity-related outcomes and PA levels in children and adolescents^35^. Bearing in mind that we did not assess PA continuously, but rather for one week before, during, and after the intervention, both groups tended to decrease their PA levels from baseline to 24 weeks. Notably, the control group increased their daily vigorous PA from 4 to 14 min per day from baseline to 12 weeks, resulting in a significant between-group difference at 12 weeks, favouring the control group. As we did not collect data to explain changes in PA patterns, the reason for this between-group difference remains unclear. It may reflect natural variation, seasonal influences, or other unmeasured factors. Contrary to our findings, a previous review of RCTs proposed exergaming as a promising approach to increase adolescents’ PA levels^36^. However, none of the six studies included in that review found a beneficial effect of exergaming on objectively assessed PA levels.^36^ In line with our findings, Madisson et al. observed gradual decreases in PA levels from baseline to 24 weeks but with no difference between groups^15^. With a mean of 11 exergaming sessions for about 46 min per session, participants in the exergaming group undertook, on average, only 21 min of exergaming per week throughout the study period. Thus, the lack of beneficial effect on PA may indicate that exergaming replaced habitual PA and was unable to motivate adolescents not already involved in regular endurance training to increase their overall PA levels. Furthermore, despite not participating in endurance training, participants were relatively active. Future research should focus on adolescents who are insufficiently active.

There were no between-group differences in blood pressure or circulating markers of cardiometabolic health, consistent with a 3-month exergaming trial in adolescent girls with overweight/obesity, which reported no improvements in blood pressure or cholesterol^14^. In contrast, a home-based exergaming trial in children with overweight/obesity showed that 24 weeks of exergaming significantly improved blood pressure and cholesterol^18^. In the present study, about half of all participants had a normal BMI, whereas all participants in the studies by Staiano et al.^14,18^ had a BMI ≥ 85th percentile. Exercise has a more pronounced effect on cardiometabolic health in those with overweight or obesity^37^. Again, the low exergaming frequency and the lack of increase in PA levels are other probable explanations for non-significant changes in blood pressure and blood markers.

## Limitations

Major limitations of our study include early trial termination, difficulties in recruitment, and a high drop-out rate, resulting in reduced statistical power for primary and secondary outcomes. The challenges during recruitment suggest a need to explore how exergames and exergaming interventions can be tailored to better appeal to the target population. We did not include a supramaximal verification phase when assessing cardiorespiratory fitness, which has been recommended for children and adolescents^38^. Another limitation is relying on two days of data for the PA assessment, despite previous investigations demonstrating that three weekdays and both weekend days are necessary to detect PA patterns with the SenseWear armband reliably^39^. In addition, we aimed to conduct the trial in a real-world setting. However, participants had to preschedule their exergaming sessions and travel to our exercise laboratory, where the exergaming platforms were located. Thus, the results could have been different if we had provided participants with more of an open-door policy and had the exergaming platforms in several locations. Furhermore, the use of bioelectrical impedance analysis to estimate body composition, the lack of standardisation of the time of day at which we undertook the body composition assessment, and the failure to confirm participants’ hydration status may have influenced our results. Although the bioelectrical impedance analyser used (InBody 720) tends to underestimate fat mass compared to dual-energy X-ray absorptiometry, it has demonstrated good reliability, and we therefore argue that its use here has limited influence on our findings^40,41^. Finally, only 2 (6.9%) participants were female.

## Conclusion

Providing adolescents not engaged in regular endurance training with access to an exergaming platform did not increase cardiorespiratory fitness but induced favourable changes in body composition. These findings should be interpreted with caution due to the small sample size, the few attended exergaming sessions, and the markedly limited representation of female participants. The observed improvements in body composition without concomitant increases in overall PA levels suggest that exergaming may motivate adolescents to make other healthy lifestyle changes, which should be assessed in future trials.

## Data Availability

Data sets generated during the current study are available from the corresponding author on reasonable request.
